# Response to: “Inclusion as Illusion: Erasing Transgender Women in Research with MSM”

**DOI:** 10.1002/jia2.25662

**Published:** 2021-01-26

**Authors:** Trevor A Crowell, Patricia E Fast, Linda‐Gail Bekker, Eduard J Sanders

**Affiliations:** ^1^ U.S. Military HIV Research Program Walter Reed Army Institute of Research Silver Spring MD USA; ^2^ Henry M. Jackson Foundation for the Advancement of Military Medicine Bethesda MD USA; ^3^ International AIDS Vaccine Initiative New York NY USA; ^4^ School of Medicine Stanford University Palo Alto CA USA; ^5^ Desmond Tutu HIV Centre University of Cape Town Cape Town South Africa; ^6^ KEMRI‐Wellcome Trust Research Programme Kilifi Kenya; ^7^ Oxford University Oxford United Kingdom

**Keywords:** transgender people, men who have sex with men, key and vulnerable populations, human rights, HIV prevention, Africa

We appreciate the letter in response to the recent *JIAS* supplement on engaging African men who have sex with men (MSM) and transgender women (TGW) who have sex with men in HIV research. The authors point out that no papers in the supplement focused solely on TGW and called for the publication of studies that explore issues affecting TGW independently from other key populations. We agree wholeheartedly with this call. In our guest editorial accompanying the supplement, we noted that TGW have historically been conflated with MSM, but are “deserving of separate consideration and tailored, rights‐affirming engagement” [1].

The supplement arose from a symposium at the R4P conference in Madrid that addressed MSM engagement in longitudinal research in Africa. Despite not being designed to do so, several studies presented there had enrolled both MSM and TGW populations. A well‐known example is the HPTN 075 study to assess MSM cohort feasibility in Kenya, Malawi and South Africa, which found that one in seven enrolled “MSM” identified as a woman or transgender female [2]. Recognizing an opportunity to help fill knowledge gaps related to this under‐studied population, the guest editors encouraged disaggregation of data by gender identity in all supplement manuscripts.

In 2019, *JIAS* published the first HIV incidence estimate among a small group of TGW from Kenya as 20.6 (95% confidence interval 6.6 to 63.8) cases per 100 person‐years (PY), and several‐fold higher than incidence estimates among MSM [3]. The recent *JIAS* supplement has added two new HIV incidence estimates among TGW from South Africa (31.0 [3.7 to 111.2]/100PY) and TGW from Nigeria (23.8 [13.6 to 39.1]/100PY), confirming an exceptional burden of new HIV infections among African TGW [4, 5]. These estimates indicate a need for urgent and targeted prevention interventions, deliberate efforts to monitor and promote uptake of pre‐exposure prophylaxis, and the development of guidelines for HIV prevention and care programming for TGW in sub‐Saharan Africa.

The JIAS supplement demonstrated that there has been progress in the engagement of African MSM and TGW. While more work involving TGW in their own right is overdue and much needed, we think that the inclusion of both TGW and MSM concurrently in research has moved the field in the right direction. Such studies also provide opportunities for comparative evaluations across key populations that underscore the unique features and needs of each population. We are excited, for example, by the planned phase III trial involving both MSM and TGW populations for an efficacy assessment of monthly long‐acting islatravir (formerly MK‐8591) for HIV prevention (IMPOWER 24), which will enroll in Kenya and other global sites.

But, as we emphasized in our guest editorial, much more must be done. There are several actionable lessons from the supplement, including a need for training programs to sensitize healthcare providers, capacity building programs for sociobehavioral researchers, and administrative support programs to build capacities of community‐based organizations representing TGW. We concur strongly that studies focused on TGW experiences in sub‐Saharan Africa are worthy of publication to inform differentiated delivery of HIV prevention and care services to this unique and vulnerable population.

## COMPETING INTERESTS

The authors report no competing interests.

## AUTHORS’ CONTRIBUTIONS

TAC, PEF, LGB and EJS conceptualized the manuscript. TAC and EJS authored the first draft of the manuscript, which was reviewed by PEF and LGB. All authors have read and approved the final manuscript for publication.
