# Corrigendum

**DOI:** 10.1002/jia2.25684

**Published:** 2021-03-05

In the article [1], the word 'seven' has been changed to 'five' in the second paragraph on e25662.

The sentence reads:

“A well‐known example is the HPTN 075 study to assess MSM cohort feasibility in Kenya, Malawi and South Africa, which found that one in seven enrolled “MSM” identified as a woman or transgender female [2]”

The sentence should have read:

“A well‐known example is the HPTN 075 study to assess MSM cohort feasibility in Kenya, Malawi and South Africa, which found that one in five enrolled “MSM” identified as a woman or transgender female [2].”

We apologize for the error.
